# Combinatorial control of temporal gene expression in the *Drosophila* wing by enhancers and core promoters

**DOI:** 10.1186/1471-2164-13-498

**Published:** 2012-09-20

**Authors:** David D O’Keefe, Sean R Thomas, Kelsey Bolin, Ellen Griggs, Bruce A Edgar, Laura A Buttitta

**Affiliations:** 1 Division of Basic Sciences, Fred Hutchinson Cancer Research Center, Seattle, WA, 98109, USA; 2 Gladstone Institute for Cardiovascular Disease, J David Gladstone Institutes, San Francisco, CA, 94158, USA; 3 German Cancer Research Center (DKFZ)-Center for Molecular Biology Heidelberg (ZMBH) Alliance, Im Neuenheimer Feld 282, Heidelberg, D-69120, Germany; 4 Molecular Cellular and Developmental Biology, University of Michigan, Ann Arbor, MI 48109, USA

**Keywords:** Drosophila melanogaster, Core promoter, Cis-regulatory sequence, Microarray, Metamorphosis, Wing Morphogenesis, Terminal differentiation, Cell cycle exit, TATA box binding protein-associated factor

## Abstract

**Background:**

The transformation of a developing epithelium into an adult structure is a complex process, which often involves coordinated changes in cell proliferation, metabolism, adhesion, and shape. To identify genetic mechanisms that control epithelial differentiation, we analyzed the temporal patterns of gene expression during metamorphosis of the *Drosophila* wing.

**Results:**

We found that a striking number of genes, approximately 50% of the *Drosophila* transcriptome, exhibited changes in expression during a time course of wing development. While cis-acting enhancer sequences clearly correlated with these changes, a stronger correlation was discovered between core-promoter types and the dynamic patterns of gene expression within this differentiating tissue. In support of the hypothesis that core-promoter type influences the dynamics of expression, expression levels of several TATA-box binding protein associated factors (TAFs) and other core promoter-associated components changed during this developmental time course, and a testes-specific TAF (tTAF) played a critical role in timing cellular differentiation within the wing.

**Conclusions:**

Our results suggest that the combinatorial control of gene expression *via* cis-acting enhancer sequences and core-promoter types, determine the complex changes in gene expression that drive morphogenesis and terminal differentiation of the *Drosophila* wing epithelium.

## Background

Within many developmental contexts, cells assemble into epithelial sheets and coordinately differentiate to form organs and tissues. This differentiation process often involves changes in cell shape, cell-cycle arrest, and the emergence of cell-type-specific features. Whereas many factors (i.e., transcription factors, signaling pathways, adhesion molecules, and cytoskeletal elements) have been identified that control individual aspects of this differentiative process, it is less clear how these divergent changes in epithelial and cell biology are temporally coordinated to generate the adult structure.

*Drosophila* wing tissue is uniquely amenable to studies that concern epithelial differentiation. During larval stages of development, presumptive wing cells proliferate within a relatively uniform, mono-layered epithelium. When the animal pupariates and metamorphosis is initiated, however, this simple epithelium rapidly transforms into a complex wing structure. The most dramatic changes associated with wing differentiation involve cell shape, as evagination and elongation of the wing pouch gives rise to the much larger wing blade. This process requires wing-cell flattening, creation of a bi-layered epithelium, and the formation of tubes (i.e., wing veins) [1,2]. Coincident with this epithelial morphogenesis, each cell executes its final cell cycle [3,4], adopts a cell-type-specific shape [5,6], and forms a polarized actin-rich wing hair [7], among other things. To identify regulatory mechanisms that initiate and coordinate the terminal differentiation of an epithelium, therefore, we have characterized the changes in gene expression that occur during a time course of *Drosophila* wing metamorphosis.

Our time-course study in the wing reveals a surprisingly complex pattern of gene regulation during metamorphosis, affecting nearly half of the genes in the *Drosophila* genome at one or more time points. We suggest that this complex regulatory pattern results from the combined influence of sequence-specific binding complexes within genetic enhancers, and temporal changes in core-promoter preference. As such, our focused analysis of a single epithelial tissue as it undergoes a dynamic developmental transition, suggests an important role for core-promoter sequences (and associated proteins) in coordinating epithelial differentiation.

## Results

**Figure 1:**
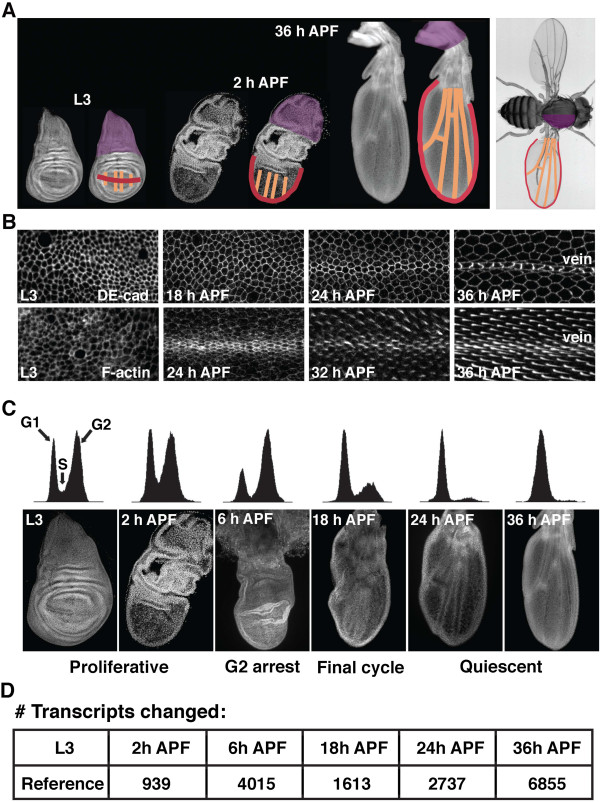
**Early stages of wing differentiation.** (**A**) To illustrate the morphogenetic process of wing-disc elongation, wing tissue from late third larval instar (L3), 2 h after puparium formation (APF), and 36 h APF was dissected and stained for DNA. The wing margin (red), wing veins (orange), and notum (purple) are indicated in developing tissue and the adult fly. (**B**) Between L3 and 36 h APF, each wing epithelial cells adopts a cell-type-specific shape, and differentiates a wing hair. Time courses of wings stained for either DE-cadherin (to visualize apical cell shape) or F-actin (to visualize hair formation) are shown. Images are centered on a presumptive wing vein. Developmental stages are indicated. (**C**) Flow cytometric analysis demonstrates changes in DNA content associated with cell-cycle exit in the wing. At L3 and 2 h APF, presumptive wing cells asynchronously proliferate. By 6 h APF, most cells in the wing temporarily arrest in the G2 phase of the cell cycle, leading to a relatively synchronized final cell cycle between 14 and 24 h APF (represented here by 18 h APF). By 24 h APF, cell proliferation is no longer detected in the wing epithelium, and nearly all cells arrest with a G1 DNA content. Dissected wing tissues stained for DNA are shown for each developmental stage. (**D**) To determine the changes in gene expression associated with wing morphogenesis and cell cycle exit, RNA was collected from six developmental time points between L3 and 36 h APF (corresponding to images shown in (C)), and microarray analysis was performed. Using L3 as a reference sample, the number of transcripts that exhibit a significantly different level of expression is listed for each time point.

Within a very short period of time (roughly 36 h at 25°C) the wing imaginal disc is transformed from a relatively simple epithelial sheet into a complex structure that resembles the adult wing (Figure 1A). To illustrate this fact, and to confirm the developmental timing of our genotype under study (*w*^*1118*^), wing tissue was dissected at several developmental time points between the late third larval instar (L3) and 36 h after puparium formation (APF). We found that wing pouch evagination began at pupariation and was clearly evident by 2 h APF (Figure 1A,C). By 6 h APF, wing elongation and hinge region constriction were occurring (Figure 1C), as described previously [8]. In addition to gross morphological changes such as these, dramatic changes in shape were also observed at the cellular level. Developing wing tissue was stained for DE-cadherin to reveal the apical shape of each cell [9]. Between 18 and 36 h APF, wing cells adopted a hexagonal shape (a process termed hexagonal packing) [6], and vein/intervein shape differences emerged [5] (Figure 1B). Each wing cell also formed a wing hair during early pupal stages [7], as revealed by F-actin localization (Figure 1B).

Between late L3 and 36 h APF, presumptive wing cells also exit the cell cycle. To precisely determine the timing of this event, we used fluorescence-activated cell sorting (FACS) analysis to characterize the temporal changes in DNA content of wing cells (Figure 1C). The wing epithelium remained proliferative through 2 h APF (i.e., high levels of G1-, G2-, and S-phase cells), but by 6 h APF many cells had temporarily arrested cycling with a G2 DNA content. This results in an *in vivo* semi-synchrony of the final cell cycle. By 18 h APF, most cells in the wing had re-entered the cell cycle and completed their final round of division (i.e., many cells were already in G1, with smaller proportions completing their final S- and G2-phases) [3,4]. By 24 h APF, over 95% of the wing was arrested in G1, and only very rare S- or M-phases were observed in the wing blade (see Additional file 1). No further cell cycling was observed in the wing blade between 24 and 36 h APF.

To capture the gene expression dynamics that drive these developmental changes, we performed a microarray analysis. RNA was isolated from late L3 wing discs (the reference sample), and from wings at 2, 6, 18, 24, and 36 h APF. For technical reasons concerning the dissection, samples from the earliest time points (L3, 2 h, and 6 h APF) contained presumptive notum tissue, whereas the later samples (18, 24, and 36 h APF) did not. This array analysis revealed a striking number of transcripts whose level of expression significantly changed during this developmental time course (Figure 1D). For example, comparison between L3 and 36 h APF yielded 6855 genes that were differentially expressed. In total, 8338 genes were differentially expressed at one or more time points.

### Gene expression dynamics during differentiation of the wing

With such a large number of differentially expressed genes, we explored computational methods of categorizing and grouping genes to identify patterns within the data. We first used self-organized mapping of genes (ordered by similarity across the five time points) to intuitively display global gene-expression dynamics. This provided a clear view of general changes in gene expression over time, but masked many discrete temporal patterns by grouping them in with the most common trends (see Additional file 2).

**Figure 2:**
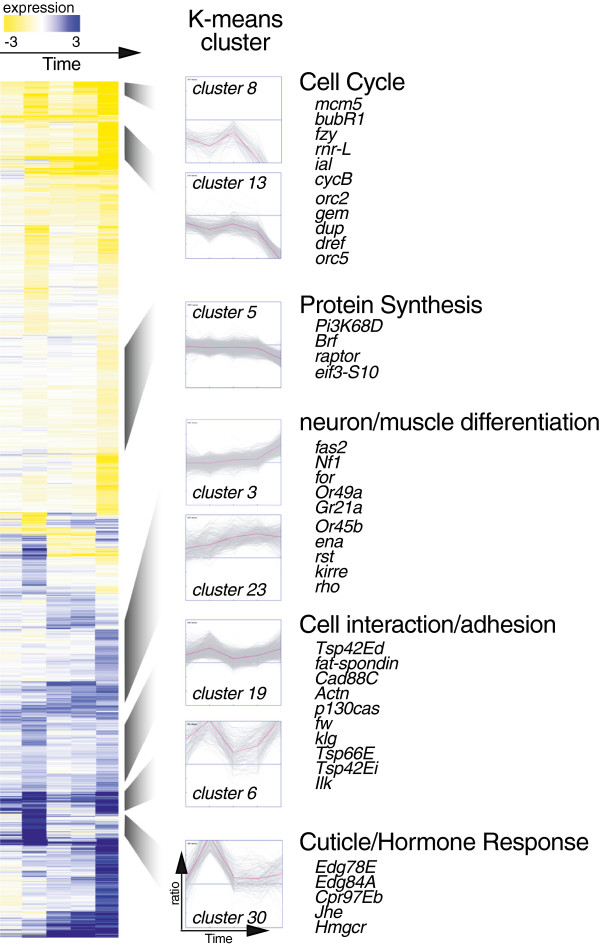
**Dramatic changes in gene expression take place during wing differentiation.** A compressed heat map shows significant global gene expression changes for 8338 genes in the wing between late L3 and 36 h APF (proliferating L3 wing tissue was used as the reference sample). Each row corresponds to a single gene and each column represents an individual time point. Expression values (log_2_) are color coded according to the legend at the top. Using k-means clustering (see Materials and Methods), genes were grouped into 30 clusters based on expression profile similarities. Expression plots are shown for selected k-means clusters and approximately aligned to corresponding regions of the heat map. Plots of all 30 k-means clusters are shown in Additional file 3. For each cluster, the normalized log_2_ expression level of each gene (grey lines) is plotted as a function of time (x-axis). The magenta line represents the average expression of all genes within a cluster. Examples of genes and enriched gene ontology terms for the selected clusters are listed at right.

We next used hierarchical clustering to organize all 8338 differentially expressed genes. From this analysis, we estimated that the dataset contained between 25 and 30 distinct temporal patterns of gene expression. Based on this information, we used the Genesis program to perform k-means clustering, imposing values of *k* that ranged from 26–40. We empirically determined that 30 clusters represented the optimal number of temporal expression patterns, without generating too many similar or overlapping clusters. Additional file 3 shows the pattern of expression for each k-means cluster and Supplemental text files 1–30 contain the transcript list and complete data for each cluster. We next hierarchically organized the genes within each k-means cluster, and ordered the clusters to generate a heat map, which represents the global changes in gene expression that occur during metamorphosis of the wing (Figure 2).

### Enhancer motifs are enriched in co-regulated clusters

We first examined gene ontology (GO) enrichment within the 30 k-means clusters to see if clusters with similar temporal patterns of expression contained functionally related genes. For this analysis 118 GO terms were used. Sorting clusters based on GO enrichment, however, did not group clusters with similar patterns of expression (see Additional file 4). During differentiation of the wing, therefore, GO terms do not accurately predict the temporal pattern of expression for a given gene.

We next asked whether similarly regulated clusters of genes share known regulatory motifs (i.e., transcription factor binding sites) within upstream enhancer regions. A database of 87 known *Drosophila* transcription factor-binding motifs (based upon the FLYREG motif set) was used for this analysis [10]. We also added several motifs to this dataset, including the mammalian E2F motif from Transfac, and custom motifs for *Drosophila* Myc, Ecdysone Receptor/Ultraspiracle complex (EcR/USP), Trithorax-like (Trl), Medea (Med), and *Drosophila* E2F. These custom motifs were obtained by performing MEME analysis on previously described target genes [11-13], and by deriving a consensus from two *Drosophila* E2F binding sites identified by Yamaguchi and colleagues [14]. The Med motif was obtained by MEME analysis of confirmed direct Decapentaplegic (Dpp) signaling target genes in the wing (DOK and LAB, manuscript in preparation). Finally, a starvation responsive E-box motif [15] was also included. See Additional file 5 for details concerning these custom motifs. Additional file 6 provides the position-specific scoring matrices (PSSM) for all motifs used for this analysis.

For each gene, 1 kb of sequence immediately upstream of the transcriptional start site (TSS) was defined as the DNA-search space. This amount of sequence provided a good compromise between capturing important regulatory elements and minimizing sequence search space, which can decrease enrichment. We empirically tested search spaces of 1, 2, and 3 kb upstream of each TSS, and found the greatest amount of motif enrichment and/or depletion when 1 kb of sequence was analyzed. This finding is consistent with published data concerning *Drosophila* regulatory elements [16,17], but certainly does not identify all binding sites within target-gene enhancers and will omit binding sites outside of the 1-kb window and in introns.

**Figure 3:**
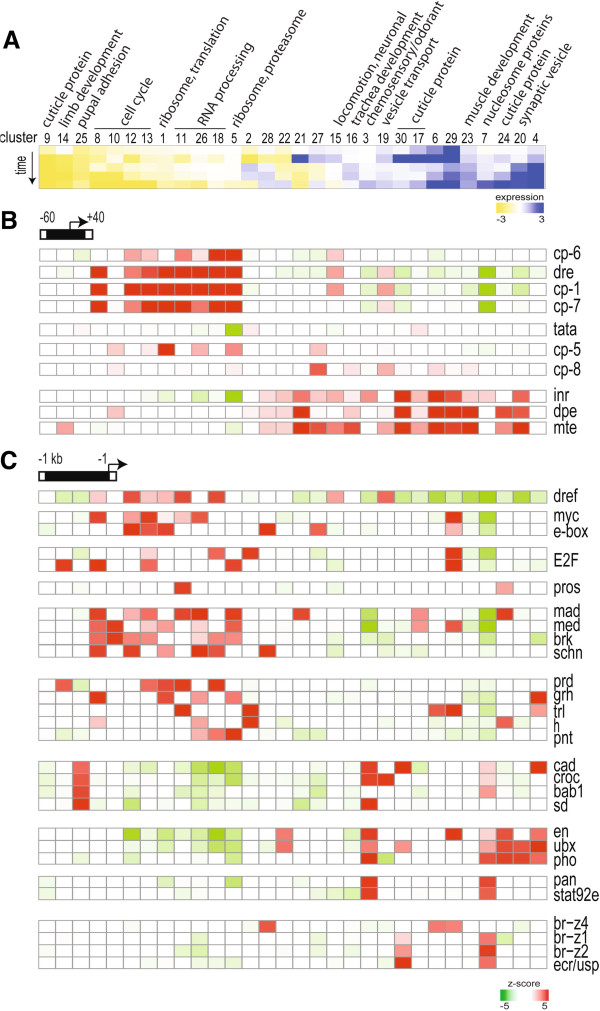
**Enrichment of core promoter types and regulatory motifs within gene expression clusters.** (**A**) Clusters were grouped based on temporal patterns of expression, which are represented by the heat map (y-axis is time). Average expression values for genes within a cluster are shown. For each cluster, the most significantly enriched gene ontology (GO) term is listed (only when p < 10^-5^). (**B**) All 30 clusters were examined for statistically significant (z-score > |3|) enrichment (red) or depletion (green) of ten core *Drosophila* promoter motifs (see Materials and Methods). The search space was defined as the 100 bp spanning the transcriptional start site (−60 to +40 bp). Certain core promoter motifs were frequently enriched in the same clusters. For example, DRE, CP-1, CP-7, and CP-6 motifs were typically enriched within a similar subset of clusters. In addition, these clusters tended to decrease expression over time. In contrast, INR, DPE, and MTE motifs were often found together in clusters that increased expression over time. TATA motifs were rarely enriched or depleted. (**C**) All 30 clusters were examined for significant enrichment or depletion of 87 known *Drosophila* transcription factor binding motifs. The search space was defined as the 1 kb immediately upstream of the transcriptional start site. A group of motifs known to regulate cell growth, protein synthesis, and the cell cycle (e.g., E2F, Myc, Dref, Mad/Med/Brinker) were enriched and frequently found together in clusters that decreased expression over time and upon cell-cycle exit. Clusters that decrease expression over time were also depleted for a group of motifs associated with tissue differentiation (e.g., Cad, Croc, En, Ubx). These differentiation-associated motifs were instead enriched in clusters that increased expression during pupal time points.

In Figure 3, the average change in gene expression for each cluster is represented by the heat map at the top, and the most significantly enriched GO term for each cluster is listed (if p < 10^-5^). Clusters were ordered based on similarities between the temporal patterns of gene expression. We found that a number of motifs known to regulate cell growth, protein synthesis, and the cell cycle (e.g., E2F, Myc, DNA-Replication-related Element Factor (Dref), Mothers against Dpp (Mad), Med, and Brinker binding motifs [18-24]) were frequently enriched in the upstream sequences of the same clusters. In addition, expression of these clusters generally declined during pupal stages of development and upon cell-cycle exit (Figure 3C). An interesting exception to this rule was cluster 29, which contains many of these same binding sites, but is characterized by increased levels of expression during pupal stages (further discussed below). We also found that motifs associated with tissue differentiation (e.g., Caudal, Crocodile, Engrailed, and Ultrabithorax binding motifs [25-32]) were significantly depleted in clusters with declining levels of expression (Figure 3C). Instead, these differentiation-associated motifs were enriched in clusters that increased expression during pupal time points. Finally, clusters that exhibited a strong peak of gene expression (log_2_ > |3|) at 6 h APF (or a dip in the case of cluster 28) were typically associated with Broad binding-site enrichment. This suggests that Broad mediates a transcriptional response to Ecdysone signaling at 6 h APF in the wing (Figure 3C). This likely mediates the G2 arrest that characterizes the 6 h APF wing, as two high-scoring potential Broad binding sites are found upstream of the gene *string,* whose downregulation mediates the temporary G2 arrest. Our data suggests that in the differentiating wing enhancer motifs are generally enriched in clusters of genes with similar temporal patterns of expression.

### Core-promoter types strongly correlate with co-regulated clusters of genes

A previous study used cell sorting coupled with serial analysis of gene expression (SAGE) to reveal a role for Dref in controlling gene-expression changes during differentiation of the *Drosophila* eye [33]. Dref is a transcription factor that acts as a component of a transcription-initiation complex containing TBP-Related Factor2 (TRF2) [34]. The Dref regulatory element (DRE) is found in upstream regulatory regions of many cell-cycle genes (e.g., *pcna*[35]), but is also one of the most common core-promoter motifs identified in *Drosophila*[36]. We found DRE enrichment in clusters of genes with declining levels of expression during terminal differentiation, which generally contained genes involved in the cell cycle and ribosomal biogenesis. This finding, combined with recent work demonstrating a role for core-promoter components in regulating gene transcription during development [37], prompted us to examine whether core-promoter types are associated with specific temporal patterns of gene expression in the differentiating wing. For each of the 30 k-means clusters, therefore, we asked whether ten core *Drosophila* promoter motifs (see Materials and Methods) were either enriched or depleted. The 100 bp spanning the TSS (from −60 to +40 bp) was defined as the DNA-search space for this analysis. Certain core-promoter (CP) motifs (i.e., DRE, CP-1, CP-7, and CP-6) were frequently found together in clusters of genes with declining levels of expression during wing differentiation. In contrast, Initiator (INR), Downstream Promoter Element (DPE), and Motif-Ten Element (MTE) motifs were often found together in clusters that increased expression over time. TATA motifs were rarely enriched or depleted in these k-means clusters (Figure 3B). In the developing wing, therefore, core-promoter type strongly correlates with the temporal patterns of gene expression, suggesting a change in core promoter usage upon terminal differentiation.

### Clusters enriched for certain motifs contain verified target genes

**Figure 4:**
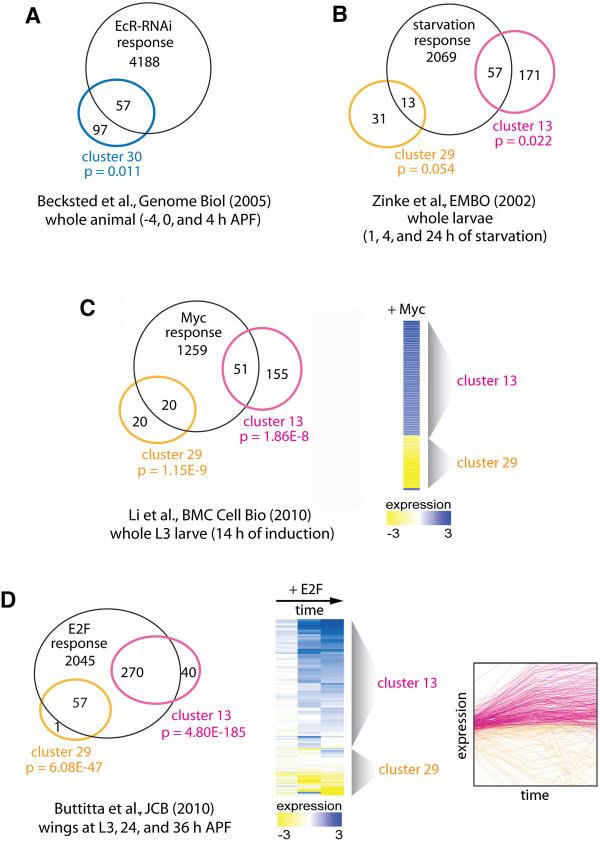
**Motif-enrichment validations.** Gene clusters with significant motif enrichment were compared to published datasets. For example, cluster 30 was enriched for EcR/USP binding sites, so genes within cluster 30 were compared to independently identified EcR/USP target genes (**A**). This analysis was repeated for clusters 29 and 13, which were both enriched for the starvation-associated motif (**B**), the Myc motif (**C**), and the Drosophila E2F motif (**D**). In every case but one, significant enrichment for independently validated target genes was observed. Confirmed Myc target genes within Cluster 13 are generally induced by Myc expression, whereas cluster 29 genes are generally repressed by Myc expression (see heat map (C)). Similarly, verified E2F targets within clusters 13 and 29 are generally upregulated and downregulated by E2F expression, respectively (see heat map and expression plot (D)).

To ask whether motif enrichment within a cluster indicates that these genes are regulated by a common factor (i.e., the transcription factor that binds the motif), we compared selected clusters to published datasets of verified target genes. For example, cluster 30 is enriched in EcR/USP motifs. To validate that this cluster represents a true EcR/USP regulon, we compared genes within this cluster to previously identified EcR/USP targets [38] (Figure 4A). Similarly, clusters 13 and 29 are enriched in the starvation-associated E-box motif, the Myc motif, and the *Drosophila* E2F motif. Genes from these two clusters were therefore compared to starvation-response genes [39], Myc target genes [15], and E2F target genes [40] (Figure 4B-D). In every case except one, significant enrichment for the independently validated target genes was observed (as measured by hypergeometic probability < 0.05). Observed enrichment was most significant when similar tissues and developmental stages were compared. This likely reflects tissue- and temporal-specific target-gene expression, as expected. Strikingly, genes within clusters 13 and 29 are enriched for both Myc and E2F motifs, yet they are characterized by different core promoter motifs, and are regulated in an opposite fashion (i.e., expression of cluster 13 and 29 decline and rise during wing differentiation, respectively). Nonetheless, the majority of genes in cluster 13 and 29 were independently validated as legitimate Myc and E2F target genes. However, nearly all cluster 13 Myc/E2F targets *increase* in response to Myc or E2F overexpression (Figure 4C,D, blue in heat maps), whereas nearly all cluster 29 genes *decrease* in response to Myc or E2F overexpression (Figure 4C,D, yellow in heat maps). Our data suggest that this dramatic switch in gene-expression behavior may depend upon the type of core promoter, although we cannot exclude the contribution of other enhancer motifs to gene expression in these clusters.

### Core-promoter type may influence gene-expression behavior

In our analysis, each transcript has a unique identifier, providing the transcription start location (TSS) for each isoform. Thus, all transcripts were treated as separate entities, allowing us to include alternate TSSs in our analysis. This raised the interesting question of whether genes with multiple TSSs that contain different core promoter types, switch core-promoter usage during differentiation.

In the *Drosophila* genome, 14% of genes are predicted to utilize multiple TSSs. Based on our analysis, a small number, 10.5% of these have identifiable DPE or DRE core promoter motifs. Of this small number of genes, there are 20 genes within the *Drosophila* genome that have a DRE at one promoter and a DPE (or another promoter motif) at an alternative TSS. In our wing dataset, nine of these genes had at least two temporally regulated transcripts that use fundamentally different core promoters. In all nine cases, the complement of promoter motifs predicted the transcript’s overall expression behavior and cluster assignment (Table 1). Transcripts from the same gene that utilized different core-promoter types did not cluster together, whereas transcripts from different genes with similar promoters frequently clustered together (e.g., clusters 3 and 23; see Table 1). These nine genes represent 22 transcripts. For 20 of the 22 alternate TSS transcripts, the predicted core promoter enrichment for the assigned cluster also correctly predicted the transcript expression pattern. For these genes, core-promoter sequences could exert a greater influence on temporal patterns of expression than more distal regulatory sequences.

**Table 1:** Cluster assignments for genes with alternate TSSs

**Gene**	**TSS type**	**Cluster**	**TSS type**	**Cluster**
Alh	Non-DPE	12	DPE	23
br	Non-DPE	9,10*	DPE	21
CG17292	Non-DPE	1	DPE	3
CG1906	DRE	5,15	Non-DRE	3
CG32158	DRE	25	Non-DRE	3
ATP-alpha	DRE	5	Non-DRE	23
Mod(mdg4)	DRE	5,18	Non-DRE	16,19*
Rtnl1	DRE	26	Non-DRE	3,9
CG4390	DRE	13	Non-DRE	3

A small number of genes in our data set employ multiple TSSs that are associated with different types of core promoters. Transcripts associated with these genes often exhibited different temporal patterns of expression (i.e., were assigned to different clusters), which was predicted by the type of promoter, in all except for two transcripts (indicated by an asterisk).

### Changes in expression of core promoter-associated factors upon terminal differentiation

**Figure 5:**
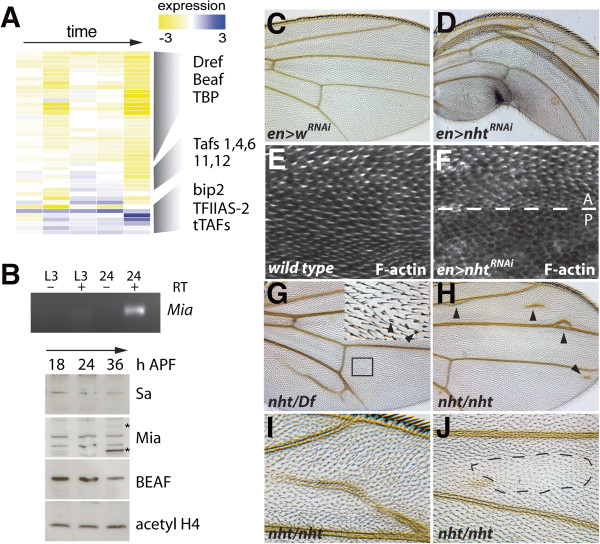
**Changes in core-promoter binding proteins may affect wing differentiation.** (**A**) Expression data from the wing developmental time course for genes that encode core promoter binding proteins, general transcription components, and TBP-associated factors (TAFs). Each row corresponds to a single gene and each column represents an individual time point. Expression values (log_2_) are color coded according to the legend at the bottom. Specific groups of genes are indicated. The complete clustering of all 72 genes is provided in Additional file 7. (**B**) RT-PCR and western blot analyses were used to examine RNA or protein levels for selected factors in pupal wing tissue. By microarray analysis *sa*, which is a tTAF, did not increase expression at the time points shown. This was verified by western blot. Levels of the tTAF Mia increased in the wing, both by RT-PCR (at 24 h APF) and western blot (at 36 h APF). Mia-specific bands at 35 and 70 kD (indicated by asterisks) are observed with anti-Mia antibody in select tissues (N. Haugen and D. Wassarman, personal communication). BEAF decreased in the wing over time. Acetylated histone H4 served as a loading control. (**C-F**) Using UAS-RNAi lines, *engrailed-Gal4* was used to inhibit either *white* (*w*), or *nht* in the posterior wing*. Tubulin-Gal80*^*TS*^ was used to limit RNAi expression from the second larval instar until eclosion. Compared to controls (C), *nht* reduction affected posterior wing growth and cuticle integrity (D). Staining for F-actin reveals developing wing hairs at 34 h APF (E). Compared to the anterior control, expression of *nht* RNAi led to delay in wing hair formation in the posterior (F). *nht*^*z5946*^ hemizygotes, exhibit ectopic vein and multiple wing hair phenotypes (**G**, inset). Two *nht* alleles in trans (*nht*^*z5347*^*/nht*^*z5946*^) at 25°C exhibit ectopic vein (indicated by arrowheads in **H**, with 20X magnification in **I**). Approximately 10% of *nht*^*z5347*^*/nht*^*z5946*^ females exhibit patches of thin, small wing hairs (outlined by dashed line in **J**).

We next examined the patterns of expression for genes that encode core promoter-binding proteins, general transcription components, and TBP-associated factors (TAFs). Expression data from the wing developmental time course was extracted for these genes, and hierarchical clustering was used to group them according to their temporal pattern of gene expression (Figure 5A, Additional file 7). Significant changes in expression were observed for core promoter element binding proteins (e.g., Dref and Boundary Element-Associated Factor (BEAF)), and TFIID components (e.g., TAFs and testes-specific TAFs (tTAFs)). Expression levels of Dref, several TAFs (2, 5, 7, 8, and 13), and an isoform of BEAF (transcript RA) that binds the DRE [41] all declined during terminal differentiation of the wing. In contrast, expression of TRF2 (a Dref partner [34]), an isoform of BEAF (transcript RB), several TAFs (3, 11, 12, and 10b), and select testes-specific TAFS (*males in absentia (mia), cannonball (can), andnohitter (nht)*) increased during the developmental time course. These observations support the hypothesis that a change in core promoter usage may be associated with terminal differentiation in the wing.

To verify the expression-array data, western-blot analysis was used to examine protein levels for selected factors in pupal-wing tissue (Figure 5B). By microarray analysis, the tTAF *spermatocyte arrest* (*sa*) did not increase expression at the time points shown. This was verified by western blot, and demonstrates selectivity in tTAF regulation within the differentiating wing (i.e., not all tTAFs were co-regulated). Levels of the tTAF Mia increased in the wing at later stages of development (both by RT-PCR and western-blot analyses), whereas total levels of BEAF decreased in the wing over time.

Using inducible RNAi transgenes, we then reduced levels of several TAFs and testes-specific TAFs (tTAFs) and examined the effect on differentiation of the wing. Because many of these genes can affect general transcription, we used the temperature-sensitive Gal80 system, in combination with wing Gal4 drivers, to express RNAi constructs from the mid-larval stage of development (L2) until eclosion (i.e., the adult stage). RNAis to specific TAFs and core promoter-associated genes resulted in disrupted wing phenotypes when expressed *via apterous-Gal4.* These included *bip3* (TAF3) and *TFIIAS-2* (Additional file 8). Employing the same experimental protocol for the tTAF *can* resulted in lethality. When the tTAF *nht* was inhibited in the posterior wing using *engrailed-Gal4* (L2 to eclosion), defects in posterior wing growth and cuticle integrity were observed (Figure 5D). Terminal differentiation in *nht* RNAi-expressing wings was assessed at the cellular level by staining these wings for F-actin at 34 h APF. Beginning at this stage of development, every cell in the wing blade forms an actin-rich wing hair, which is an early differentiation event. Expression of *nht* RNAi in the posterior wing led to a cell-autonomous delay in wing-hair formation and terminal differentiation (Figure 5F), revealing an unexpected potential somatic role for *nht*.

In further support of a function for *nht* in the wing, we noted that flies hemizygous for the EMS loss of function alleles *nht*^*z5946*^ or *nht*^*z5947*^[42] were sub-viable and 50% of escapers were developmentally delayed by 24 hours during the pupal stage. Nearly all *nht*^*z5946*^ hemizygous adults exhibited ectopic wing vein phenotypes (Figure 5G) and 10% exhibited multiple wing hair phenotypes (Figure 5G, inset). Fifty percent of *nht*^*z5347*^*/nht*^*z5946*^ transheterozygous males exhibited a mild ectopic vein phenotype in the anterior wing and 10% of females raised at 25°C exhibited ectopic vein (Figure 5H-I) and occasional patches of small, thin wing-hairs (Figure 5J).

Vein refinement involves the coordinated action of multiple signaling pathways and a developmental delay can affect the vein the refinement and limitation process. Other genotypes exhibiting wing hair developmental delay also exhibit multiple wing hairs or thin wing hair phenotypes [43], further suggesting a developmental delay in the wing occurs due to loss of *nht*. This result, combined with the promoter/motif analysis, suggests that a change in TAFs and core-promoter type preference may play an important role in regulating gene expression during differentiation of the wing. In total, our results indicate that the combinatorial influence of enhancer occupancy and core-promoter usage shapes the spatial and temporal patterns of gene expression that drive epithelial differentiation.

## Discussion

### Dramatic gene-expression changes during metamorphosis of the wing

Previous studies with whole animals estimated that 10% of the genome is differentially regulated during metamorphosis [44-46]. Many tissue-specific changes in gene expression are likely obscured by using whole animals however, as Li and White found that expression levels for 17% of the genes in the genome are significantly affected in at least one of five tissues during the developmental transition into metamorphosis [47]. Strikingly, our analysis of wing tissue during metamorphosis revealed significant changes in expression for ~50% of the genome (8338 genes). This likely reflects the wide range of developmental and cellular processes that are involved in differentiation of the wing (e.g., cell adhesion, cytoskeletal remodeling, apoptosis, neurogenesis, and cuticle formation). Changes to the cell cycle are particularly dynamic during this developmental time period (late L3 to 36 h APF), as initially proliferative cells temporary arrest in G2, synchronously execute a final cell cycle, and permanently exit the cell cycle in G1 (Figure 1). Our results indicate that such a breadth of developmental changes ultimately depends upon the modulation of several thousand genes. Our data suggests that this dynamic program of gene-expression regulation involves combined inputs from both enhancer sequences and the type of core promoter. Furthermore, this suggests that only a small proportion of genes will truly fit the definition of “constitutive” or “housekeeping” when examining dynamic developmental processes across multiple tissues.

### Temporal gene-expression patterns correlate most strongly with core-promoter types

In the differentiating wing, gene expression patterns were not highly correlated with GO term enrichment, i.e., functionally related genes were not co-regulated. Instead, shared upstream-regulatory motifs and the type of core promoter were more accurate predictors of a gene’s temporal pattern of expression. These findings are in contrast to a recent analysis, which suggests that co-regulated genes in *Drosophila* are more likely to share GO terms than transcription factor binding sites [48]. These authors based their conclusions on gene-expression data from whole organisms, an approach that may have masked tissue-specific patterns that are necessary to find shared transcription factor binding sites. Instead our analysis is consistent with the findings of FitzGerald et al., [49] which found core promoter type strongly correlated with gene expression in embryos vs. adults. We also found that enrichment for a particular type of core promoter more accurately predicted a gene’s temporal pattern of expression than enrichment for any particular enhancer motif within 1 kb of the TSS. Comparing the same tissue at multiple developmental time points, therefore, has revealed an unappreciated level of transcriptional regulation that depends upon the core-promoter type. As the wing differentiates, we found a shift in expression away from genes with DRE, and certain core promoter types (CP-1, -6, and −7), toward genes that use INR, DPE, and MTE promoter elements. This trend even held true for genes with multiple TSSs containing different core promoters, where in nearly every case we observed a shift in TSS usage during differentiation (Table 1). This shift may reflect changes in TAF and core promoter associated factors during terminal differentiation, and is consistent with studies that have found wing differentiation functions for additional components of the basal transcription machinery (summarized in Additional file 9) [50-53].

### The type of core promoter may dictate target-gene induction or repression in response to Myc and E2F

For general enhancer analysis, promoters such as the *heat-shock protein* (*HSP*) 70 TATA promoter are thought to be sufficient. Validation of the reporter-gene expression pattern is typically performed at a limited number of pre-selected time points, and reporters that recapitulate the expected pattern are chosen. Our data suggest however, that such constructs may miss important aspects of temporal regulation that are not captured by an arbitrary choice of core promoter.

Two gene clusters in our dataset (cluster 13 and 29) were enriched for both E2F and Myc binding sites, and regulated known E2F and/or Myc target genes [15,40]. However, most genes within cluster 13 were induced by dMyc/dE2F1, whereas most genes within cluster 29 genes were repressed by dMyc/dE2F1 activity (Figure 4). The latter represents a non-canonical response, as these factors primarily activate transcription when overexpressed. We identified a difference in core-promoter sequences between these two clusters, as cluster-13 genes were enriched for DRE, CP-1, -6, and −7 sequences, whereas cluster-29 genes were enriched for DPE, MTE, and INR sequences. This finding, together with our observation that the expression of genes encoding for core promoter-associated proteins were temporally regulated in the differentiating wing, suggests that differences in core-promoter type may underlie the non-canonical behavior of these E2F and Myc target genes. Most studies concerning E2F regulation use either well-characterized endogenous DRE-containing promoters [54], cell-cycle gene promoters that contain DREs [55], or engineered constructs with an *HSP70* TATA-type promoter [56]. Our data suggest that there may be another level of E2F (and possibly Myc) transcriptional regulation that is missed by using reporters such as these. This idea is supported by findings that an enhancer can be functionally linked to a specific core promoter in *Drosophila*[57], and that certain factors can stimulate DPE-dependent transcription and inhibit TATA-dependent transcription [58]. Future studies that involve reporter constructs with different types of core promoters (like those generated in [57] and [59]) will be necessary to verify and characterize this new level of regulation.

### A requirement for a tTAF in wing terminal differentiation

A previous report indicated that expression of *nht* (a tTAF) is restricted to the male germline [42]. It was surprising to find therefore, that *nht* may play a role in the proper timing of terminal differentiation in the pupal wing. However, *nht* expression had only been examined in embryos (0–24 h after egg deposition (AED)) and adults [42]. Newer modEncode transcriptome data found that *nht* transcription is undetectable until pupal stages of development [60], whereas the adult wing contains very few viable cells, due to developmentally regulated apoptosis that occurs immediately after eclosion [61]. We found that *nht*, as well as several other tTAFs including *mia* and *sa*, are expressed in the pupal wing. These results are based on microarray, RT-PCR and western blotting analyses, where possible (Figure 5). Our tTAF expression data do not contradict previous findings, therefore, but instead reveal somatic expression at a different stage of development.

*nht* mutants are viable but male sterile, consistent with their well-defined role in terminal differentiation of spermatocytes [42]. Although wing phenotypes associated with *nht* loss-of-function have not been reported, we used an inducible RNAi construct to inhibit *nht* function in the pupal wing. This acute knockdown approach revealed a dramatic wing hair differentiation phenotype that may be partially masked in *nht* mutants by compensatory changes in other TAFs. We also observed wing vein and abnormal wing-hair phenotypes in *nht* mutants (Figure 5). This genetic data demonstrates an unexpected role for *nht* in wing development. Our results suggest that non-canonical TAFs may play an important role in terminal differentiation of somatic tissues as well [62,63], including TAFs that were previously thought to be germline specific.

## **Conclusions**

The dynamic changes in gene expression during *Drosophila* wing terminal differentiation encompass approximately 50% of the protein coding genes in the genome, and are directed by combinatorial inputs from both enhancer sequences and the core-promoter type at individual genes. Our results suggest that a change in core promoter preference, likely mediated by a shift in expression of core promoter binding proteins, plays a much more significant role in modulating gene expression during metamorphosis than previously recognized.

## Methods

### Microarrays

For pupal wing microarray hybridizations, 10 pupal wings were dissected from *w*^*1118*^ animals (Bloomington Stock #3605) that had been raised on standard cornmeal/molasses media under uncrowded conditions (less than 50 larvae per vial) at 25°C. Wandering L3 was defined as wandering larvae at the gut half-empty stage (approximately 110 h AED). All pupae were staged from 0 h APF, defined as stiff white pre-pupae (approximately 120 h AED ± 20 min at 25°C). RNA was isolated from dissected tissues using Trizol (Invitrogen, Grand Island, NY), and cleaned using the RNAEasy Kit (Qiagen, Valencia, CA). RNA integrity was confirmed *via* agarose gel. Using 500–1,000 ng input RNA for each reaction, cDNA synthesis was performed with one subsequent round of T7-dependent linear RNA amplification, using the commercially available Message AmpTM kit from Ambion [40]. Amplified RNA was quantified via nanodrop, and its integrity confirmed *via* Bioanalyzer (Agilent, Santa Clara, CA). According to Nimblegen protocols (Madison, WI), 10 mg of RNA was subsequently labeled in a cDNA synthesis reaction and hybridized to Nimblegen 4-plex 60-mer *Drosophila* expression arrays ( http://www.nimblegen.com). Hybridizations were repeated four times with independently obtained biological replicates to ensure maximal confidence in data reproducibility. NimbleScan software was used for array scanning and quantile normalization [64]. Gene calls were generated using the Robust Multichip Average (RMA) algorithm [65]. All arrays in this study were normalized together. MA plots of the array data, showing a linear relationship between intensity and average intensity post-normalization, is provided (see Additional file 10). Statistically significant changes in gene expression (adjusted p < 0.05) were determined using ANOVA. The mean fold change for genes with significant changes was 2.2. Approximately half of the genes exhibited fold changes > 2, and half exhibited less than a 2-fold change. We also compared our results to previously published RT-PCR and microarray data where similar pupal wing stages were possible (Additional file 11) [66].

The data discussed in this publication have been deposited in NCBI’s Gene Expression Omnibus. The GEO Series accession number is GSE36015.

#### *Drosophila* lines

The following lines were used in this study: *w*^*1118*^ (Bloomington Stock, #3605), *nht*^*z5347*^*/nht*^*z5946*^ , Df(2 L)A263 (Bloomington Stocks #25159, 6062 and 25160), *engrailed*-Gal4, UAS-GFP; *tub*-Gal80^TS^ (*engrailed*-Gal4, UAS-GFP from [67], *tub*-Gal80^TS^ from [5]), UAS-*nht*^RNAi^ (Vienna *Drosophila* RNAi Collection, stock #10351). For experiments using the temperature-sensitive (TS) Gal80 system, animals were shifted from 18°C to 28°C at the indicated times.

### Gene expression clusters

Genes differentially expressed at one or more time points compared to the wandering L3 reference sample (> 1.5-fold) were grouped into 30 clusters using the k-means clustering algorithm with Euclidean distance in the Genesis program ( http://genome.tugraz.at). Hierarchical clustering was also performed in Genesis to generate heat maps showing transcript changes (log_2_ ratio of expression compared to L3 reference) for all transcripts with a fold-change ≥ 1.3 (> log_2_ ± 0.4) at one or more time points. For the gene expression heat map of 30 k-means clusters shown in Figure 3, the average expression for all genes within the indicated cluster at the indicated time point is shown.

### Western blotting, RT-PCR, and histology

Western blots using 10 wild-type *Drosophila* pupal wings at the indicated stages were performed as described [68]. Blots were incubated with primary antibodies directed against acetylated histone H4 (1:2000; Millipore, Billerica, MA), BEAF (1:200; Developmental Studies Hybridoma Bank (DSHB)), Sa (1:100; kindly provided by Dr. M. Fuller), Mia (1:100; a kind gift from Dr. D. Wassarman). Appropriate horse-radish peroxidase-conjugated secondary antibodies were used at 1:2000 and detected using enhanced chemiluminescence reagents (GE Healthcare, Piscataway, NJ). For RT-PCRs, 10 dissected wings of the indicated stages were homogenized in Trizol for RNA isolation and clean up using the RNAeasy kit (Qiagen) with DNAse treatment to remove genomic DNA. RNA was quantified using a nanodrop, integrity was confirmed using gel electrophoresis, and 500 ng was used for cDNA synthesis *via* the oligo-dT primed, Superscript III kit (Invitrogen). PCR was carried out using 1/10 of the cDNA reaction using Go Taq (Promega, Madison, WI), with 35 amplification cycles. Primers for *mia* are available upon request. Mock reactions without RT (−RT in Figure 5) were carried out to ensure PCR products were not from genomic DNA contamination.

Pupal wings of the indicated stages were fixed with 4% paraformaldehyde in phosphate-buffered saline (PBS) and stained using a primary antibody directed against DE-cadherin (DSHB) and an Alexa-fluor 568 secondary antibody as described [5]. Pupal wings were stained for nuclei using Hoechst 33258 at 0.5 μg/mL in PBS, or F-actin using Rhodamine-labeled phalloidin diluted 1:200 in PBS (Molecular Probes). For F-actin staining, wings were not exposed to detergent. Confocal images of pupal wings were taken using a Zeiss LSM510 confocal microscope at 20× and Zeiss AIM software.

Adult wings were dehydrated in 100% ethanol, placed in methyl salicylate (Sigma, St. Louis, MO) for 10 min, and permanently mounted and flattened in Canada balsam and methyl salicylate (1:1) (Sigma). Adult wings were photographed under brightfield conditions on a Leitz Orthoplan2 at 10-20× magnification, using a Nikon DS-Vi1 color camera and Nikon NIS Elements software.

### Flow cytometry

Twenty wild-type *Drosophila* wings of the indicated stages were dissected, dissociated in a trypsin/PBS solution and stained for DNA using Hoechst 33342 as described [69]. Dissociated cells were analyzed using a FACS Vantage Cytometer (BD) with CellQuest software. At least 20,000 cells were measured to generate cell-cycle histograms.

### Motif analysis

Fimo [70] was used (at p < 0.0001) to identify the locations of relevant DNA-binding motifs (Additional file 6 includes all PSSMs used. Additional file 5 shows custom motifs added to the FLYREG dataset). Core promoter (CP) motif PSSMs have been described [36]. For all genes, Fimo scans were performed independently at the core promoter (−60 to +40 bp), and across the extended promoter region (−1 kb to −1 bp) of all genes.

To identify motifs that were enriched or depleted in given gene clusters, the sum of all motif occurrences for each cluster was calculated. Permutation tests were then performed to determine the significance of seeing that many motif occurrences at random in a cluster with ‘n’ genes where ‘n’ equals the number of genes for each cluster. For each permutation test, ‘n’ genes were randomly chosen to form a random cluster. The sum of each given motif’s occurrence in a randomly selected set was recorded. One thousand such permutations were performed to calculate the mean and standard deviation of motif occurrences in randomly selected clusters of ‘n’ genes. From the distribution of motif occurrences, a z-score was calculated to express the enrichment or depletion of a given motif in a real cluster of genes compared with randomly-chosen clusters of equal size. A threshold z-score of |3| was chosen as significant for enrichment or depletion of motifs. Permutation tests were performed to calculate z-scores for each cluster and motif combination.

### Gene ontology analysis

Gene ontology terms were obtained from FlyBase ( http://www.flybase.org). For each cluster of genes, the enrichment of functional terms associated with that cluster was calculated using the hypergeometric distribution, which calculates the probability of randomly drawing ‘b’ genes with a given functional term from among all ‘N’ genes in the genome, given that there were ‘n’ genes in the cluster (the number of draws from the genome) and ‘B’ total genes with that annotation in the entire genome.

### Validation of cluster motif enrichment

Hypergeometric probabilities were calculated using the hypergeometric distribution. In our case: (1) the population size equals the total number of genes on our microarray platform, (2) the number of successes in the population is the total number of genes that are represented in both our dataset and the published dataset under comparison, (3) the sample size is the total number of genes in each cluster (for which there is data in the published dataset under comparison), and (4) the number of successes in the sample equals the total number of genes in that cluster that are validated as significantly changed by the treatment or transcription factor under study in the published dataset under comparison. A p-value less than 0.05 indicated a significant overlap.

## Abbreviations

ANOVA: Analysis of variance; APF: After puparium formation; CP: Core promoter; DRE: DNA replication-related element; FACS: Fluorescence activated cell sorting; FIMO: Find individual motif occurrences; GO: Gene ontology; L3: Larval instar stage 3; MEME: Multiple em for motif elicitation; PBS: Phosphate-buffered saline; PSSM-: Position specific scoring matrix; RNAi: RNA interference; SAGE: Serial analysis of gene expression; TAFs: TBP-associated factors; TBP: TATA binding protein; TS: Temperature sensitive; TSS: Transcriptional start site; tTAF: Testes-specific TAF.

## Competing interests

The authors declare that they have no competing interests.

## Authors’ contributions

DDO, LAB, and BAE conceived of the project. DDO, SRT, and LAB performed the experiments, analyzed the data, and wrote the manuscript. KB and EG assisted with experiments in Figure 5. All authors read and approved the final manuscript.

## Supplementary Material

Additional file 1 **The final cell cycle in the*Drosophila*wing.** Pupal wings at the indicated hours after pupa formation (h APF) were either fixed, stained and photographed as described [67] or exposed to Bromodeoxyuridine (BrdU) for S-phase labeling for 1 h prior to fixation, as described [67]. Primaray antibodies directed against Elav (1:100, DSHB) to detect postmitotic neurons; BrdU (1:100, Becton Dickinson); *Drosophila* Cyclin A (CycA) to detect cells in G2; and phospho-Ser10-histone H3 (PH3) (1:4,000, Upstate) to detect mitotic chromatin, were used. Wings showed very few to no S-phases or mitoses after 24 h APF. G2 phases, as indicated by high levels of CycA, were only observed in the anterior margin of the wing at 24 h APF. Click here for file

Additional file 2 **Gene-expression changes in the wing during terminal differentiation.** A gene expression heat map shows log_2_ changes in gene expression at the indicated time points (wing tissue from the late third larval instar was used as the reference sample). Genes were organized into 10 groups by the Self-organized Mapping (SOM) clustering method using the program GENESIS and organized by similarity. Each row corresponds to a single gene and each column represents an individual time point. Expression values are color coded according to the legend at the top. Click here for file

Additional file 3 **K-means clustering of genes that exhibit changes in expression during wing differentiation.** K-means clustering with Euclidean distance (*via* the Genesis program) was used to group genes into 30 clusters based on temporal similarities in their expression profiles (see Materials and Methods). The normalized log_2_ expression level for each gene in the cluster is plotted as a function of time (x-axis) in grey. The magenta line represents the average expression of all the genes within the cluster. Click here for file

Additional file 4 **GO-term enrichments are not shared by co-regulated clusters.** The 30 k-means clusters were sorted based on enrichment for 118 listed gene ontology (GO) terms. Each column represents a single cluster and each row represents a single GO term. The presence of a dot indicates enrichment for the indicated term of at least p < 0.001. The size of the dot is inversely correlated with the p-value. At the bottom is shown a gene-expression heat map for the wing developmental time course. Expression levels represent the average of all genes within each cluster. Developmental stages are indicated. Click here for file

Additional file 5 **Additional motifs for enhancer analysis.** We added custom motifs to the FLYREG motif set for the factors listed. Motifs for Myc, EcR/USP, Trl were obtained by performing MEME analysis on target genes described in [11,12] and [13], respectively. A *Drosophila* E2F motif was derived *via* consensus between two identified *Drosophila* E2F binding sites [6]. The generated Trl motif was compared to the previously obtained Trl motif in the FLYREG database *via* TOMTOM, which resulted in the indicated q-value. The starvation-responsive E-box motif is from [15]. The custom Medea motif was obtained by MEME analysis of confirmed Dpp target genes in the wing (DOK and LAB, manuscript in preparation). Click here for file

Additional file 6 Excel file containing position-specific scoring matrices (PSSM) for all motifs used in this analysis. Click here for file

Additional file 7 **Expression data for core promoter binding proteins, general transcription components, and TBP-associated factors (TAFs).** Hierarchical clustering (Genesis software) was used to sort 72 genes with predicted functions in basal transcription processes, based on their temporal patterns of gene expression. Each row corresponds to a single gene and each column represents an individual time point. Expression values (log_2_) are color coded according to the legend at the top. Click here for file

Additional file 8 **Manipulation of specific general transcription components and TBP-associated factors affects terminal differentiation in the wing.** Using *apterous-Gal4* in combination with *tubulin-Gal80*^*TS*^, RNAi transgenes for *TFIIAS-2, bip, beaf*, and *nht* (from VDRC) were expressed in the dorsal wing from the second larval instar until eclosion. The same experimental protocol was also used to overexpress *Dref*. Compared to controls (*w*^*RNAi*^), effects in wing growth, wing elongation, vein formation, and cuticle integrity were observed when these genes were manipulated. Two *nht* EMS alleles were put in trans to assess effect of *nht* loss of function on wing development. Fifty percent of *nht*^*z5347*^*/nht*^*z5946*^ males exhibited a mild ectopic vein phenotype (arrowheads). Two examples are shown. Panel at right is a 20x magnification of the left panel. Click here for file

Additional file 9 Published studies showing wing differentiation-specific functions for components of the basal transcriptional machinery. Click here for file

Additional file 10 **MA plots of normalized microarray data.** NimbleScan software was used for array scanning and quantile normalization. All arrays in this study were normalized together. MA plots of the array data, show a roughly linear relationship between intensity and average intensity post-normalization. Click here for file

Additional file 11 **Comparisons between our microarray data and published expression fold changes(*via*qPCR) concerning similar stages of wing development.** Click here for file
