# Supplementary figures and images for: Combinatorial control of temporal gene expression in the Drosophila wing by enhancers and core promoters
Source: BMC Genomics. 2012 Sep 20;13:498. doi: 10.1186/1471-2164-13-498 (PMC3641971; doi:10.1186/1471-2164-13-498)

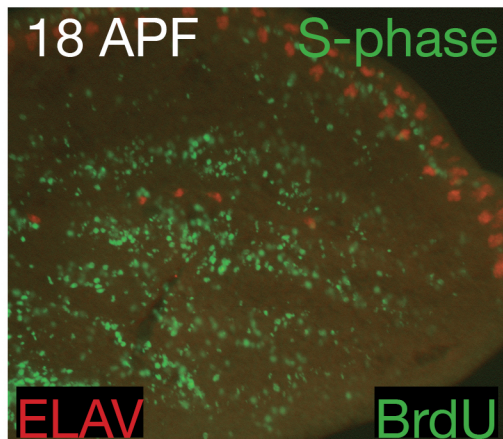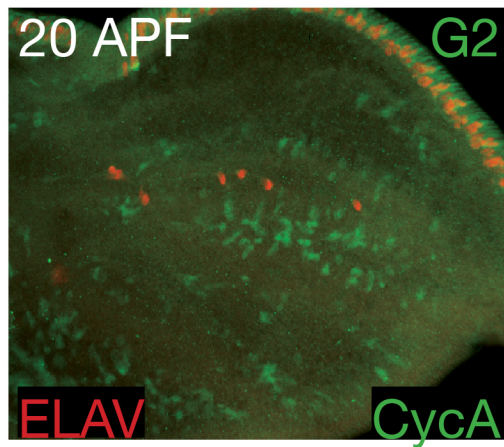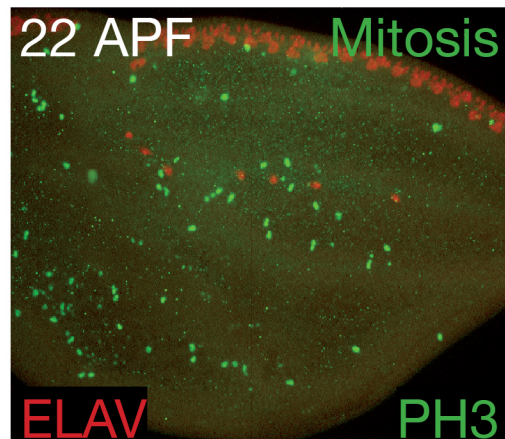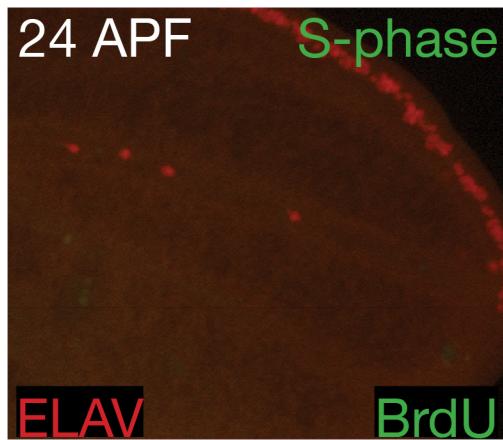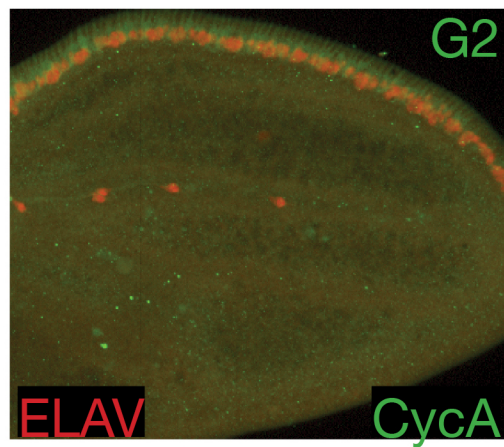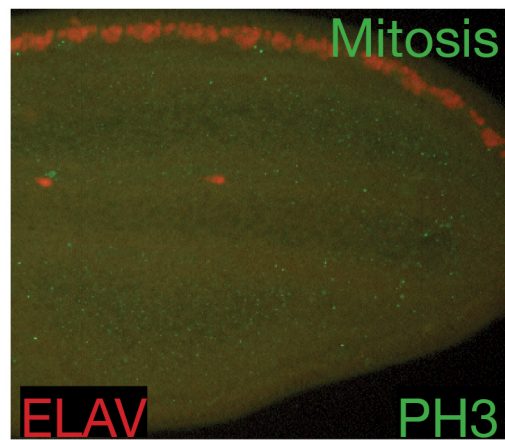

Supplement: Additional file 1 — The final cell cycle in theDrosophilawing. Pupal wings at the indicated hours after pupa formation (h APF) were either fixed, stained and photographed as described [67] or exposed to Bromodeoxyuridine (BrdU) for S-phase labeling for 1 h prior to fixation, as described [67]. Primaray antibodies directed against Elav (1:100, DSHB) to detect postmitotic neurons; BrdU (1:100, Becton Dickinson); Drosophila Cyclin A (CycA) to detect cells in G2; and phospho-Ser10-histone H3 (PH3) (1:4,000, Upstate) to detect mitotic chromatin, were used. Wings showed very few to no S-phases or mitoses after 24 h APF. G2 phases, as indicated by high levels of CycA, were only observed in the anterior margin of the wing at 24 h APF. [file 1471-2164-13-498-S1.pdf]

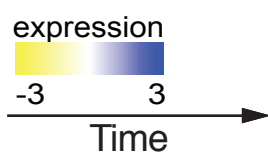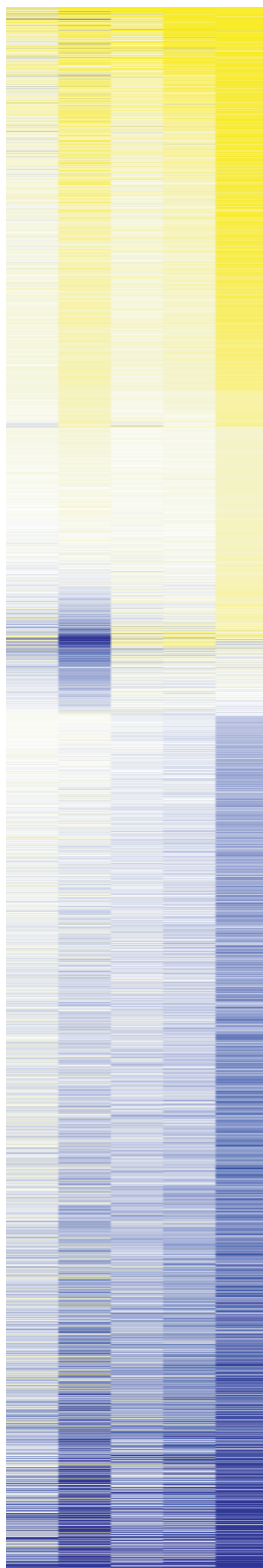

Supplement: Additional file 2 — Gene-expression changes in the wing during terminal differentiation. A gene expression heat map shows log2 changes in gene expression at the indicated time points (wing tissue from the late third larval instar was used as the reference sample). Genes were organized into 10 groups by the Self-organized Mapping (SOM) clustering method using the program GENESIS and organized by similarity. Each row corresponds to a single gene and each column represents an individual time point. Expression values are color coded according to the legend at the top. [file 1471-2164-13-498-S2.pdf]

# K-means clustering of Wing Timecourse Expression Data

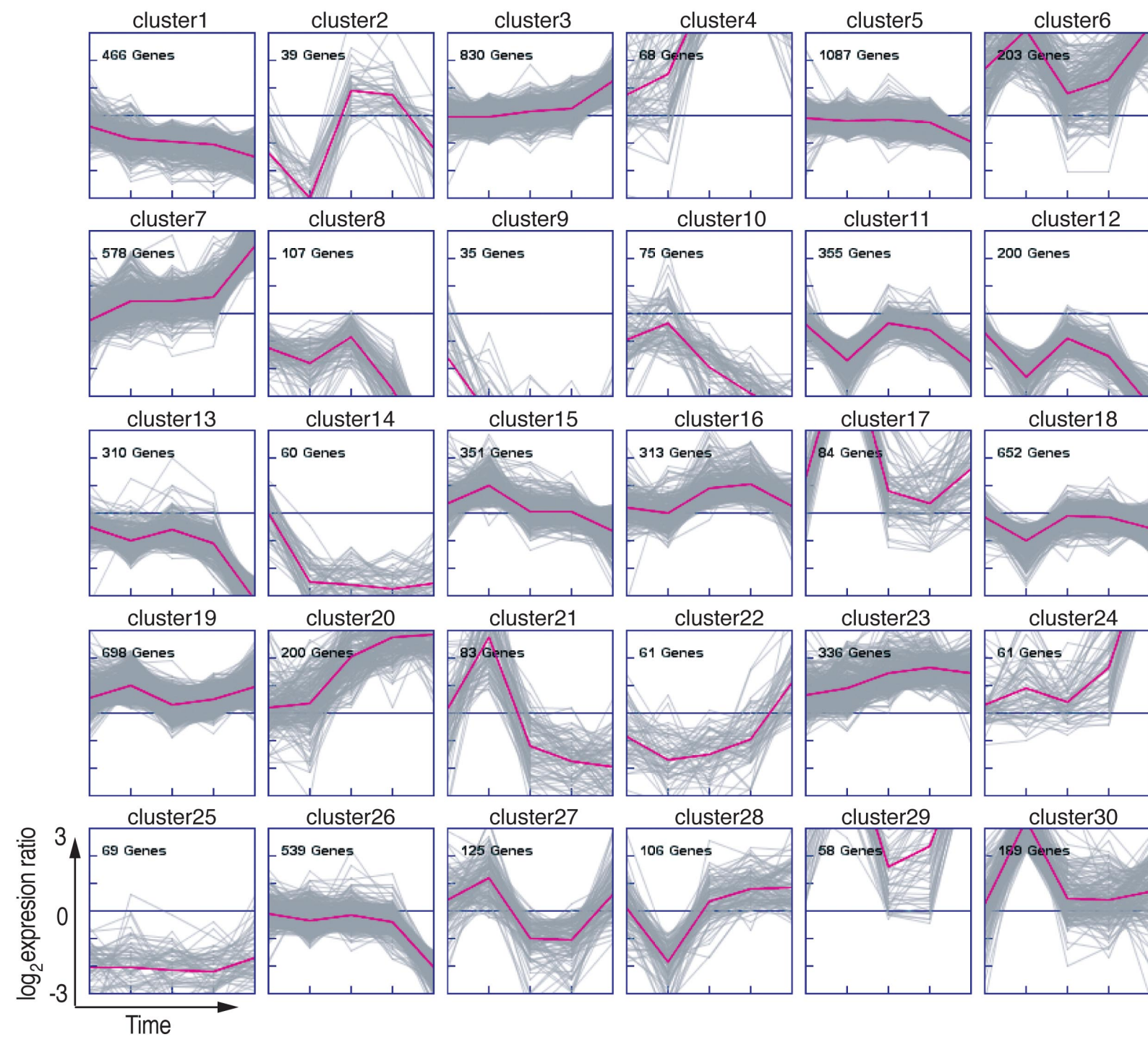

Supplement: Additional file 3 — K-means clustering of genes that exhibit changes in expression during wing differentiation. K-means clustering with Euclidean distance (via the Genesis program) was used to group genes into 30 clusters based on temporal similarities in their expression profiles (see Materials and Methods). The normalized log2 expression level for each gene in the cluster is plotted as a function of time (x-axis) in grey. The magenta line represents the average expression of all the genes within the cluster. [file 1471-2164-13-498-S3.pdf]

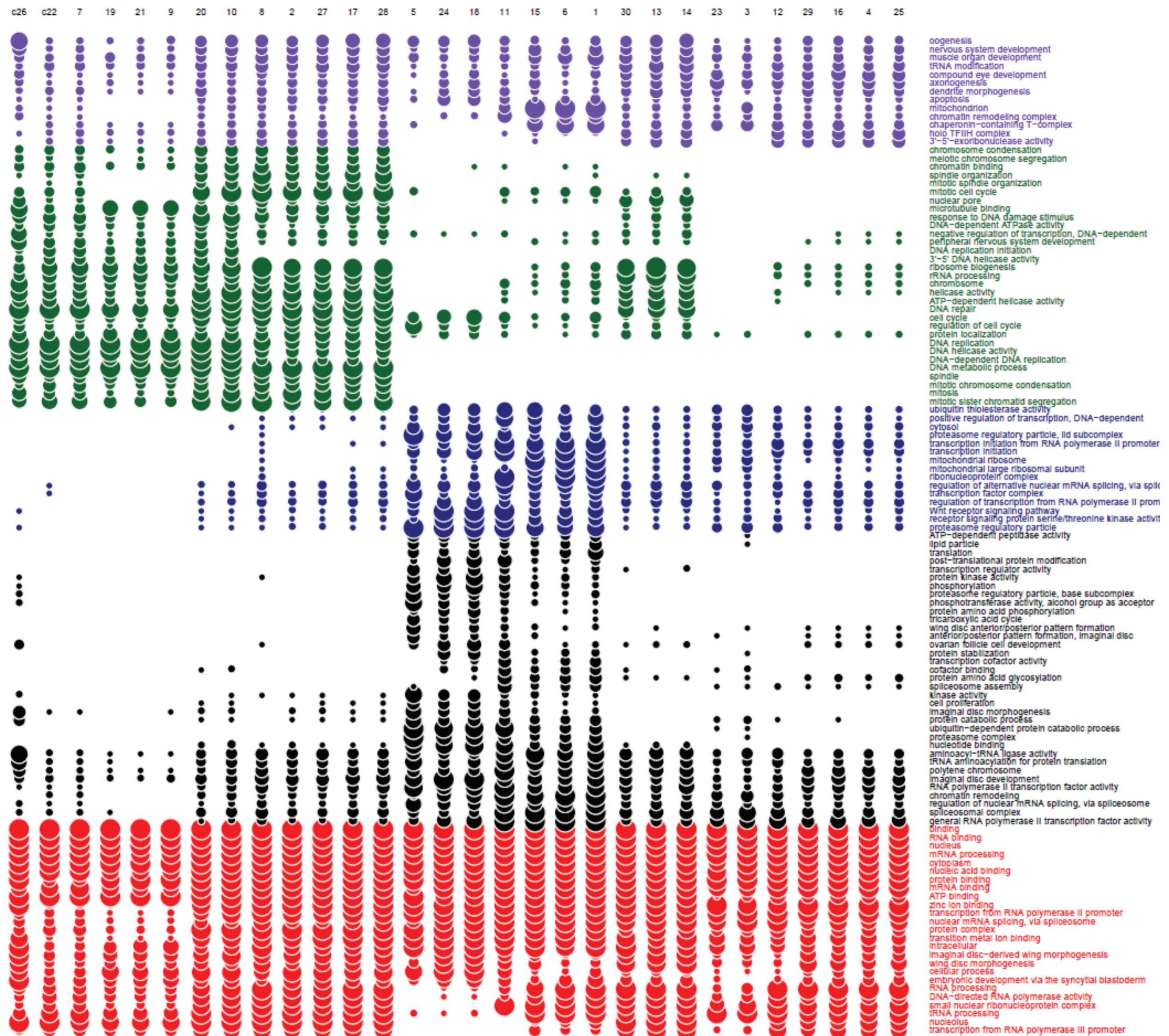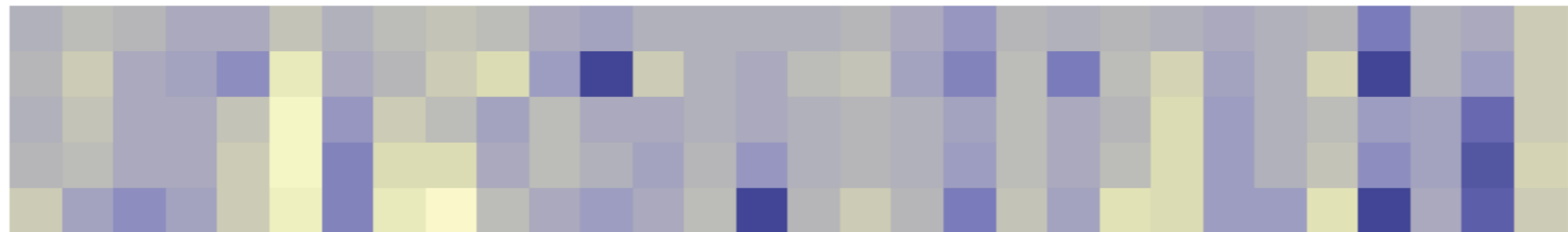

gene expression (blue=enriched)

Supplement: Additional file 4 — GO-term enrichments are not shared by co-regulated clusters. The 30 k-means clusters were sorted based on enrichment for 118 listed gene ontology (GO) terms. Each column represents a single cluster and each row represents a single GO term. The presence of a dot indicates enrichment for the indicated term of at least p < 0.001. The size of the dot is inversely correlated with the p-value. At the bottom is shown a gene-expression heat map for the wing developmental time course. Expression levels represent the average of all genes within each cluster. Developmental stages are indicated. [file 1471-2164-13-498-S4.pdf]

Additional Figure 8

Microarray data for basal transcription associated factors.

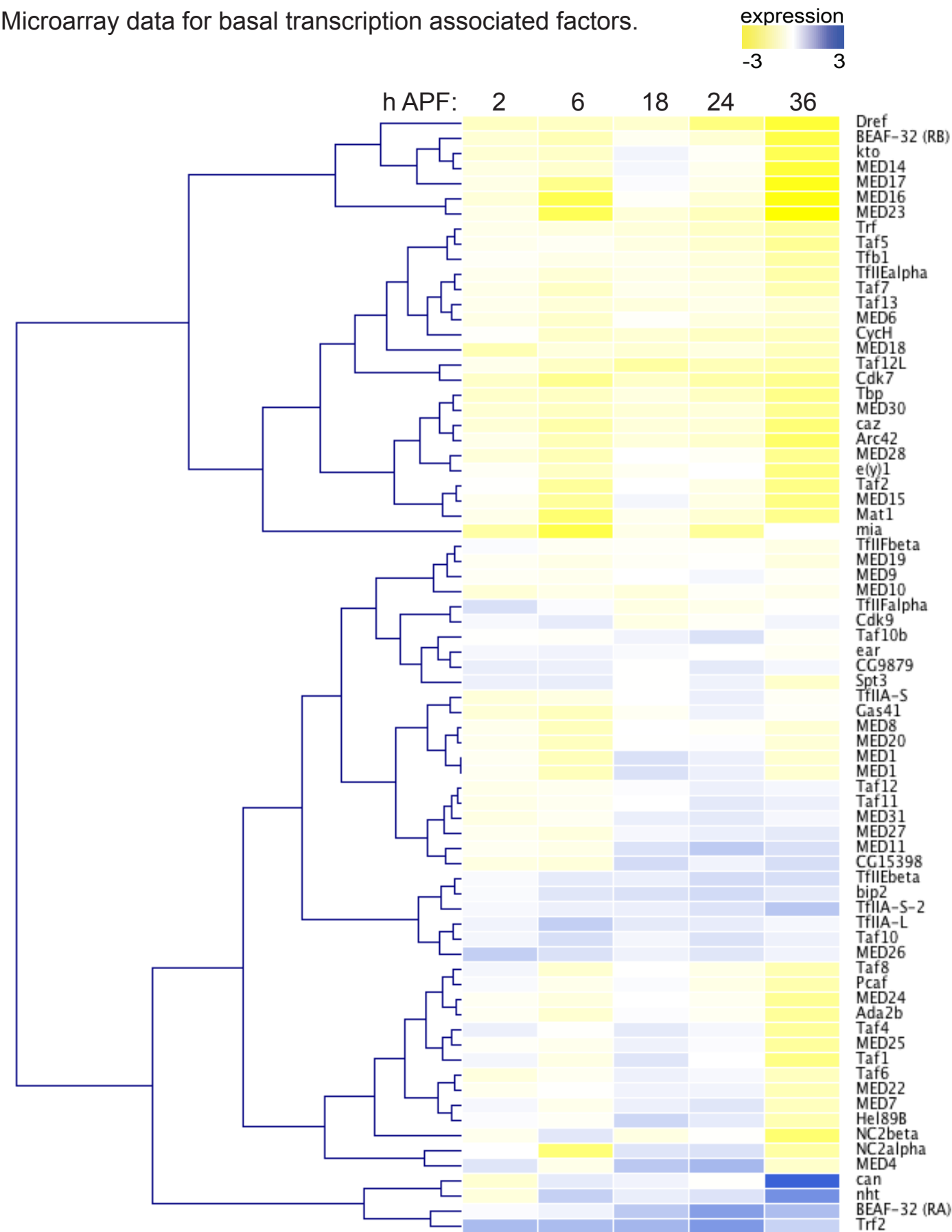

Supplement: Additional file 7 — Expression data for core promoter binding proteins, general transcription components, and TBP-associated factors (TAFs). Hierarchical clustering (Genesis software) was used to sort 72 genes with predicted functions in basal transcription processes, based on their temporal patterns of gene expression. Each row corresponds to a single gene and each column represents an individual time point. Expression values (log2) are color coded according to the legend at the top. [file 1471-2164-13-498-S7.pdf]

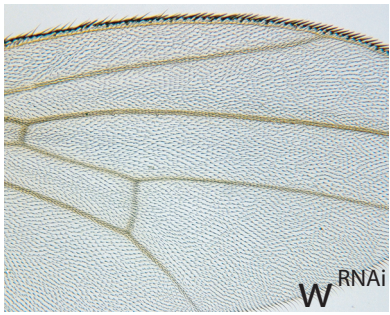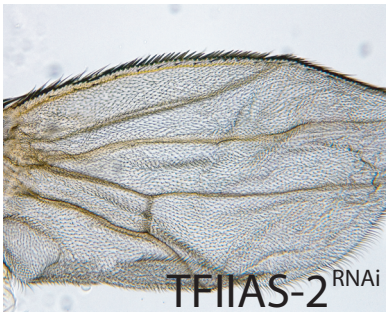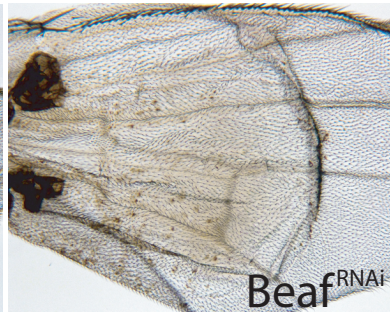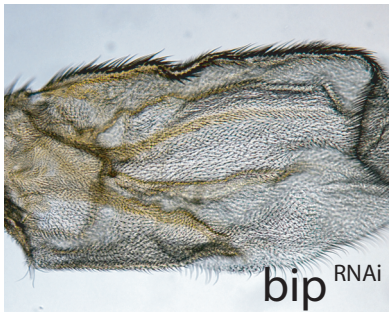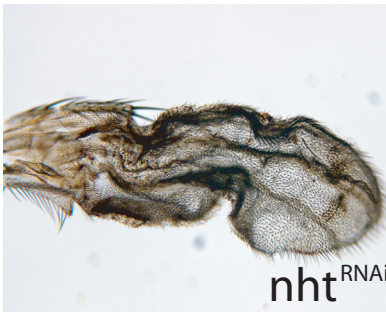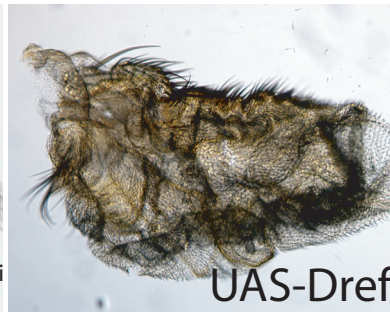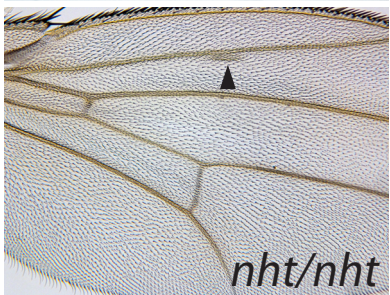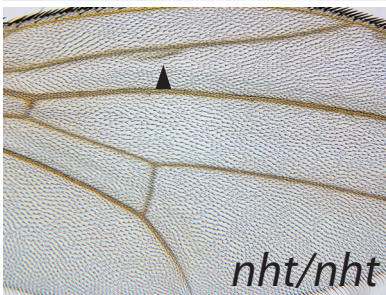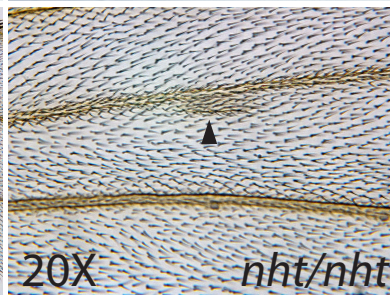

Supplement: Additional file 8 — Manipulation of specific general transcription components and TBP-associated factors affects terminal differentiation in the wing. Using apterous-Gal4 in combination with tubulin-Gal80TS, RNAi transgenes for TFIIAS-2, bip, beaf, and nht (from VDRC) were expressed in the dorsal wing from the second larval instar until eclosion. The same experimental protocol was also used to overexpress Dref. Compared to controls (wRNAi), effects in wing growth, wing elongation, vein formation, and cuticle integrity were observed when these genes were manipulated. Two nht EMS alleles were put in trans to assess effect of nht loss of function on wing development. Fifty percent of nhtz5347/nhtz5946 males exhibited a mild ectopic vein phenotype (arrowheads). Two examples are shown. Panel at right is a 20x magnification of the left panel. [file 1471-2164-13-498-S8.pdf]
