# Supplementary material for: Combinatorial control of temporal gene expression in the Drosophila wing by enhancers and core promoters
Source: BMC Genomics. 2012 Sep 20;13:498. doi: 10.1186/1471-2164-13-498 (PMC3641971; doi:10.1186/1471-2164-13-498)
Supplement: Additional file 5 — Additional motifs for enhancer analysis. We added custom motifs to the FLYREG motif set for the factors listed. Motifs for Myc, EcR/USP, Trl were obtained by performing MEME analysis on target genes described in [11,12] and [13], respectively. A Drosophila E2F motif was derived via consensus between two identified Drosophila E2F binding sites [6]. The generated Trl motif was compared to the previously obtained Trl motif in the FLYREG database via TOMTOM, which resulted in the indicated q-value. The starvation-responsive E-box motif is from [15]. The custom Medea motif was obtained by MEME analysis of confirmed Dpp target genes in the wing (DOK and LAB, manuscript in preparation). [file 1471-2164-13-498-S5.pdf]

Additional Motifs

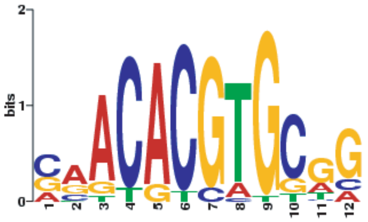

myc

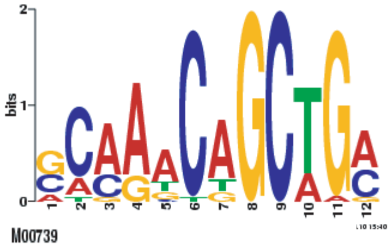

starvation response

E-box

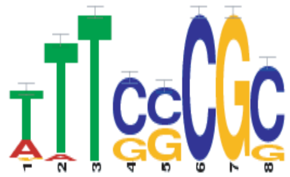

E2F

transfac

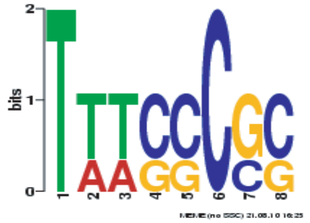

E2F

Drosophila

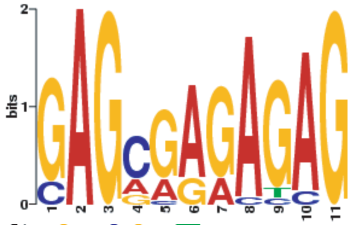

Trl

q.val. 1.9e-3

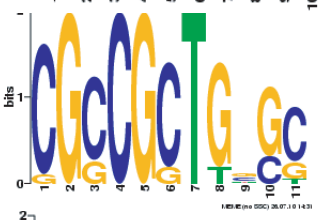

med

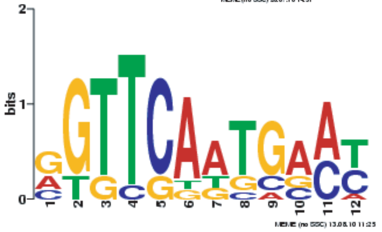

EcR/USP
