# Supplementary material for: Combinatorial control of temporal gene expression in the Drosophila wing by enhancers and core promoters
Source: BMC Genomics. 2012 Sep 20;13:498. doi: 10.1186/1471-2164-13-498 (PMC3641971; doi:10.1186/1471-2164-13-498)
Supplement: Additional file 9 — Published studies showing wing differentiation-specific functions for components of the basal transcriptional machinery. [file 1471-2164-13-498-S9.docx]

**Additional Table 2 Published studies showing wing differentiation-specific functions for components of the basal transcriptional machinery**

| gene | reference |
| --- | --- |
| *kto* | Terriente-Felix et al., 2010 |
| *Med10* | Terriente-Felix et al., 2010 |
| *Med20* | Terriente-Felix et al., 2010 |
| *Med25* | Terriente-Felix et al., 2010 |
| *Med27* | Terriente-Felix et al., 2010 |
| *Med30* | Terriente-Felix et al., 2010 |
| *Med15* | Terriente-Felix et al., 2010 |
| *Taf6* | Aoyagi and Wassarman 2001 |
| *Taf1* | Wassarman et al., 2000 |
| *Bip2* | Prince et al., 2008 |
