# Supplementary material for: Combinatorial control of temporal gene expression in the Drosophila wing by enhancers and core promoters
Source: BMC Genomics. 2012 Sep 20;13:498. doi: 10.1186/1471-2164-13-498 (PMC3641971; doi:10.1186/1471-2164-13-498)
Supplement: Additional file 10 — MA plots of normalized microarray data. NimbleScan software was used for array scanning and quantile normalization. All arrays in this study were normalized together. MA plots of the array data, show a roughly linear relationship between intensity and average intensity post-normalization. [file 1471-2164-13-498-S10.pdf]

Additional Figure 7

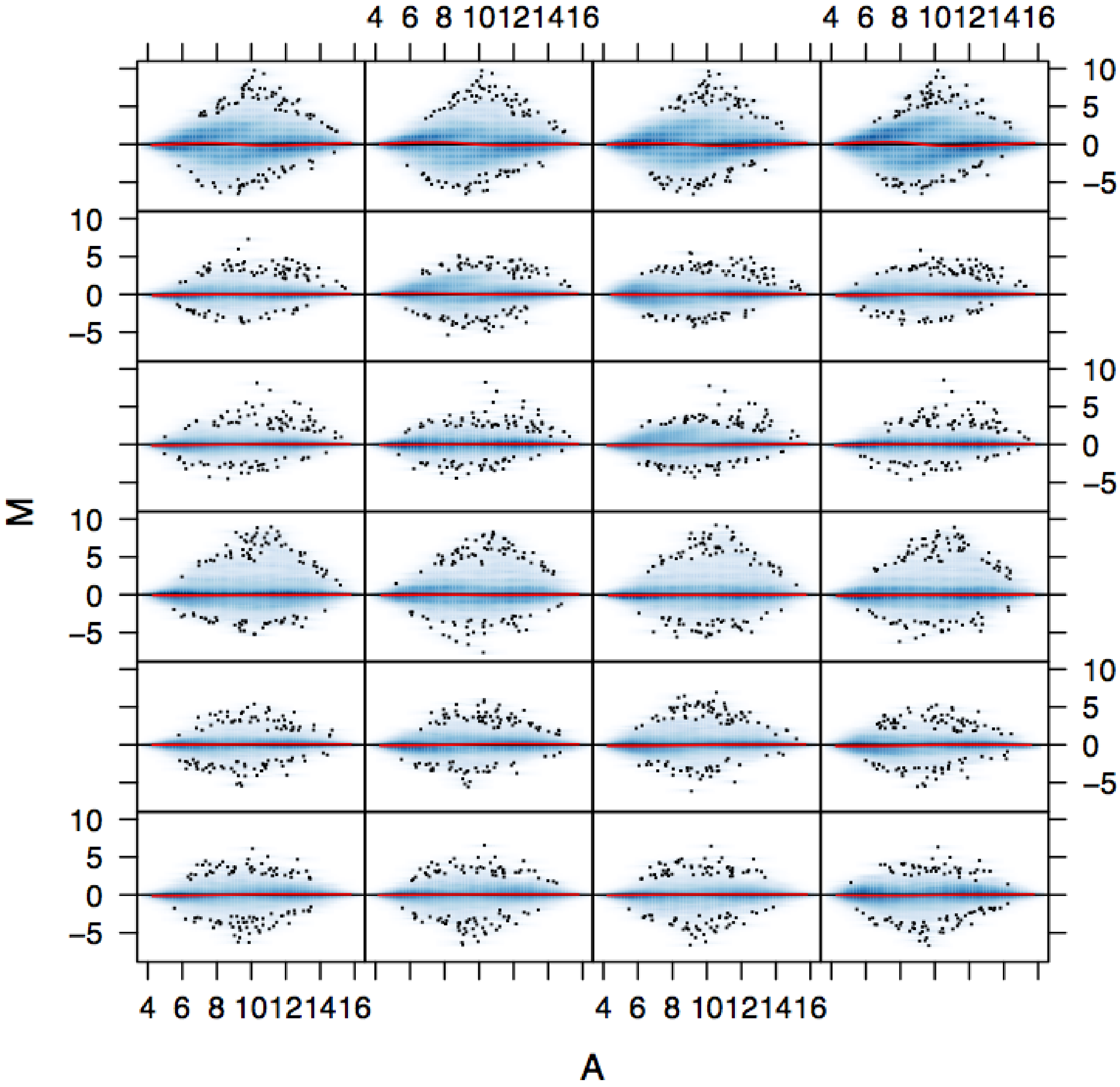

MA PLots of normalized array data. Rows indicate timepoints from late (top) to early (bottom) of L3, 2hr APF, 6 hr APF, 24h APF, and 36h APF (top).
Columns are biological replicates for each timepoint.
