# Supplementary material for: Combinatorial control of temporal gene expression in the Drosophila wing by enhancers and core promoters
Source: BMC Genomics. 2012 Sep 20;13:498. doi: 10.1186/1471-2164-13-498 (PMC3641971; doi:10.1186/1471-2164-13-498)
Supplement: Additional file 11 — Comparisons between our microarray data and published expression fold changes(viaqPCR) concerning similar stages of wing development. [file 1471-2164-13-498-S11.docx]

**Additional Table 3 Comparisons between our microarray data and published expression fold changes (*via* qPCR) concerning similar stages of wing development**

| **Gene** | **40/24 h APF [66]** | **36/24 h APF (our array)** | **36/24 h APF [68]** |
| --- | --- | --- | --- |
| *CG13209A* | 6.87 | 4.92 |  |
| *CG1869* | 56.1 | 2.64 |  |
| *CG8213* | 393.4 | 68.6 |  |
| *Fkbp13* | 5.31 | 3.25 |  |
| *dy* | 760.1 | 78.8 |  |
| *f* | 1.72 | 8.50 |  |
| *stg* | 1.57 | -6.01 | -3.58 |
